# Identification of SOX9 Interaction Sites in the Genome of Chondrocytes

**DOI:** 10.1371/journal.pone.0010113

**Published:** 2010-04-09

**Authors:** Chun-do Oh, Sankar N. Maity, Jing-Fang Lu, Jiexin Zhang, Shoudan Liang, Francoise Coustry, Benoit de Crombrugghe, Hideyo Yasuda

**Affiliations:** 1 Department of Genetics, M.D. Anderson Cancer Center, The University of Texas, Houston, Texas, United States of America; 2 Department of Bioinformatics and Computational Biology, M.D. Anderson Cancer Center, The University of Texas, Houston, Texas, United States of America; National Institute on Aging (NIA), National Institutes of Health (NIH), United States of America

## Abstract

**Background:**

Our previous work has provided strong evidence that the transcription factor SOX9 is completely needed for chondrogenic differentiation and cartilage formation acting as a “master switch” in this differentiation. Heterozygous mutations in *SOX9* cause campomelic dysplasia, a severe skeletal dysmorphology syndrome in humans characterized by a generalized hypoplasia of endochondral bones. To obtain insights into the logic used by SOX9 to control a network of target genes in chondrocytes, we performed a ChIP-on-chip experiment using SOX9 antibodies.

**Methodology/Principal Findings:**

The ChIP DNA was hybridized to a microarray, which covered 80 genes, many of which are involved in chondrocyte differentiation. Hybridization peaks were detected in a series of cartilage extracellular matrix (ECM) genes including *Col2a1*, *Col11a2*, *Aggrecan* and *Cdrap* as well as in genes for specific transcription factors and signaling molecules. Our results also showed SOX9 interaction sites in genes that code for proteins that enhance the transcriptional activity of SOX9. Interestingly, a strong SOX9 signal was also observed in genes such as *Col1a1* and *Osx*, whose expression is strongly down regulated in chondrocytes but is high in osteoblasts. In the *Col2a1* gene, in addition to an interaction site on a previously identified enhancer in intron 1, another strong interaction site was seen in intron 6. This site is free of nucleosomes specifically in chondrocytes suggesting an important role of this site on *Col2a1* transcription regulation by SOX9.

**Conclusions/Significance:**

Our results provide a broad understanding of the strategies used by a “master” transcription factor of differentiation in control of the genetic program of chondrocytes.

## Introduction

The transcription factor SOX9 plays a critical role in cell fate decisions of a discrete number of cell types [1]–[4]. Heterozygous mutations in *Sox9* cause Campomelic Dysplasia (CD), a generalized disease of cartilage characterized by hypoplasia of endochondral bones [5], [6]. Conditional inactivation of the *Sox9* gene at various times during mouse limb development also demonstrated that SOX9 is necessary for mesenchymal condensations, for the commitment to the chondrocyte fate at the time when the chondrocyte and osteoblast lineages segregate from a common progenitor, and for the overt differentiation of these cells into chondrocytes. SOX9 thus acts as a master regulator of chondrocyte differentiation [7], [8]. Chondrogenesis is associated with activation of a repertoire of cartilage-specific ECM genes. In several of these genes, chondrocyte-specific enhancers have been identified. These enhancers contain binding sites for SOX9 and mutations in these sites strongly decrease or abolish the activity of these enhancers in transfection experiments and in transgenic mice [9]–[12]. SOX9 functions as a transcription factor by recognizing a specific heptameric DNA sequence (A/T)(A/T)CAA(A/T)G through its high mobility group (HMG)-box domain. The characterization of SOX9 dimerization mutants identified in some CD patients, suggests that SOX9 binds to an inverted repeat of the heptameric sequence and that this dimeric binding is necessary for the SOX9-dependent expression of chondrocyte-related genes [13]. Chondrogenesis is also controlled by a complex interplay of signaling molecules among which some target either the expression or the activity of SOX9. Whereas IL-1 and TNF α inhibit its expression [14], FGF signaling increases its expression and its activity [15]; Wnt/β-catenin also inhibits its activity and expression [16], whereas PTHrP increases its activity [17]. In order to determine whether genes involved in cartilage function and regulation are direct targets of SOX9 in the genome of chondrocytes, and to examine patterns of SOX9 interactions with the chromatin of these genes in these cells, we have used a chromatin immunoprecipitation (ChIP)-on-chip approach [18]. Our study, which identified many new direct targets of SOX9 as well as potential binding sites for SOX9 in these genes, provides new insights in the strategies used by SOX9 in the control of chondrogenesis. In addition, characterization of a novel SOX9-dependent activator segment in intron 6 of *Col2a1* revealed that this site appears to be depleted of nucleosomes.

## Results

### Construction of the array for ChIP-on-chip

As chromatin source for ChIP-on-chip experiments, we used rat chondrosarcoma cells (RCS cells), because these cells display many chondrogenic characteristics including secretion of specific cartilage ECM proteins and high contents of SOX9, SOX5 and SOX6 [19]. When the expression levels of several mRNAs in RCS cells were compared to those in Rat-2 fibroblast cells (Figure S1 and Table S3), the transcription factors, SOX9, SOX5 and SOX6 were expressed at higher levels in RCS cells compared to Rat-2 fibroblast cells. The mRNAs for matrix proteins specific for chondrocytes including *Col2a1*, *Col11a1*, *Matrilin-1*, *Aggrecan*, *Syndecan-3*, *Cdrap*, *Fibromodulin* and *Prelp* were also highly expressed in RCS cells. On the other hand, the *Col1a1* gene was expressed at high level in Rat-2 cells but was not expressed in RCS cells. These results in addition to the previously reported data [19] indicate that RCS cells maintain a chondrocyte specific phenotype.

Table 1 lists the 80 genes that were placed on the high density custom oligonucleotide array. Each gene was covered from 15 kb 5′of exon 1 to 10 kb 3′of the last exon with overlapping 50 mers, the overlap consisting of 22 bases with the preceding oligonucleotide. Oligonucleotides for both sense and antisense strands of each gene were printed on the array. The sheared, cross-linked chromatin fragments of rat chondrosarcoma (RCS) cells [19] were immunoprecipitated with either SOX9 antibodies or with non-specific IgGs. The DNAs of both anti-SOX9 and non-specific IgG precipitated chromatin fragments were PCR amplified, labeled and then hybridized to identical oligonucleotide arrays. In selecting the genes to be placed on the array, our major rationale was to examine genes that had been shown to be expressed in cartilage. This list of genes shown in Table 1 is divided into three groups. One corresponds to genes for extracellular matrix (ECM) components. We also placed several genes for small leucine-rich proteins on the array, since mutations in some of these genes lead to osteoarthritis [20]. This first group further includes genes for *AdamTS 5* [21], *MMP9* and *13* [22], and also *Cathepsin B* [23], all of which are involved in ECM turnover. Other genes of group 1 also include the chains of type I collagen, which are not expressed in chondrocytes but are prominent in both SOX9-expressing mesenchymal precursors and in osteoblasts [24]. The second group was composed of genes for transcription factors including genes that have a major role in chondrocyte differentiation, such as *Sox9*, *Sox5* and *Sox6*, and in cartilage development such as *Prx* [25], *PGC-1α* [26], *TCF4* [27], *Lef1* [28], *β-catenin* [29], *Stat1* [30] and *TIP60* [31]. This group also included genes with a role in the osteoblasts differentiation, namely *Runx2* [32] and *Osterix (Osx)* [33]. This group further included genes for transcription factors expressed in an altogether different lineage such as *MyoD* [34] and *Myogenin* [35], which are not expressed in chondrocytes. The third group consisted of genes for signaling molecules involved in limb development or in various steps of chondrogenesis. These included genes for *Integrins α11* [36], *BMP2* and *4* [37], the BMP antagonists *Noggin* [37] and *Chordin* [38], *TGFβ3* [39], different *Wnts* [29], *Patched* [40], *Ihh* [41], *Shh* [42], *Smoothened* [43], *VEGF* [44], *Ctgf* [45], *Egf* and *Egfr* [46], *Igf1* and *Igf2r* [47], *PTHrP* [48], *Grb10* [49], *TNF α* [50], *IL1α* [51], *PKC and p38* [52], *Fgfr3* [53], *PKA* [54], *Ncam* [55], *α-Catenin* [56], and *CD44* [57].

**Table 1:** List of genes placed on the array.

Gens for Extracellular Matrix Proteins		Genes for Transcription Factors	Genes for Signaling Molecules	
Col2a1	Matrilin 1	Sox9	BMP2	CTGF
Col9a2	Matrilin 3	Sox5	BMP4	TGF-β1
Col11a1	Matrilin 4	Sox6	Noggin	TGF-β3
Col11a2	Cdrap	TCF4	Chordin	Grb10
Col3a1	Cthrc1	Lef1	Wnt3a	Integrin-α11
Col1a1	Adam-TS5	Stat1	Wnt5a	NCAM
Col1a2	MMP9	Tip60	Wnt7a	ERK1
Aggrecan	MMP13	PGC-1α	Wnt9	ERK2
Syndecan-3	Cathepsin B	β-catenin	Shh	p38
Fibronectin	Chondroadherin	Runx2	Patched	PKA
Linkprotein	Lubricin	Osx	Smoothened	PKCα
Asporin	Prelp	Myogenin	PTHrP	Arhj
Biglycan	Osteoadherin	MyoD	FGFR3	CD44
Decorin	Epiphycan		EGF	Asb4
Fibromodulin	Osteoglycin		EGFR	α-catenin
Lumican	Opticin		IGF1	TNF-α
			IGF2r	IL-1α
			VEGF	

### Criteria for positive SOX9 interaction sites

Because the sheared chromatin fragments had a size between 1 and 1.5 kb, the peaks of hybridization were relatively broad. To be counted as SOX9 interaction sites the peaks had to have similar heights at identical locations on both the forward and reverse DNA strand lanes. A so called smoothened lane was generated by subtracting the corresponding non-specific IgG hybridization signals from each SOX9 signal followed by averaging the two subtracted signals. Overall, except for a few genes discussed below, peaks, which on the “smoothened” lane had a cut off above the base 2 logarithm of 1.5 were further examined.

### Location of SOX9 interaction peaks

**Figure 1:**
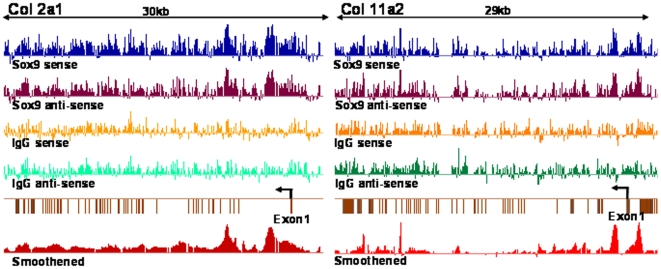
Identification of SOX9 interaction sites in rat *Col2a1* and *Col11a2* genes by ChIP-on-chip. ChIP-on chip using SOX9 antibody showed specific hybridization peaks, which were further modified by background subtraction to form smoothened peaks shown at the bottom of each panel. Exons are shown as solid bars. In the *Col2a1* gene (left panel), we clearly detected two peaks, one in intron 1 and the other in intron 6. In the *Col11a2* gene (right panel), we clearly detected two peaks, one in the promoter region and the other in intron 1. Exons shown in the right side of exon 1 in *Col11a2* were those in the adjacent gene.

Figure 1 illustrates examples of actual hybridization results for two genes in which SOX9 interaction sites were identified. Two clear peaks were identified in *Col2a1*. The center of the peak located in intron 1 corresponds to a previously identified chondrocyte-specific enhancer in this gene [9]. The other peak is located in intron 6 and the characterization of this SOX9 interaction site will be presented in a subsequent section, that is SOX9 binds to intron 6 of *Col2a1*. Two SOX9 interaction sites were also identified in the *Col11a2* gene. The centers of these hybridization peaks, one located in the promoter and the other in intron 1, correspond to previously characterized chondrocyte-specific enhancers [10]. Identification of clear ChIP hybridization peaks centered at previously characterized chondrocyte-specific enhancers suggested that the hybridization peaks in other genes on the array might also correspond to *bona fide* specific SOX9 interaction sites. A total of 55 peaks with a cut off >log_2_ 1.5 were identified in 30 genes. These genes and the locations of these peaks within these genes are listed in Table 2. A majority of the peaks were located either in promoters or in 5′ introns, rarely in regions 3′ to the gene. Most genes for cartilage-specific ECM components, contain at least two SOX9 interaction peaks. These include, in addition to *Col2a1* and *Col11a2* [10], *Col9a2* [58], *Syndecan-3* [59], *Aggrecan* [12], *Epiphycan* [60], *Chondroadherin* [61] and *Biglycan* [62]. A few genes, which have smoothened peaks below but close to the log_2_ 1.5 cut off, are, however, also likely to be direct targets of SOX9 and are likely to be part of the genetic program of chondrocytes controlled by SOX9. These genes are listed in a separate section of Table 2. Interestingly the *Col1a1* gene, which is not expressed in chondrocytes but is prominently expressed in mesenchymal precursors and in osteoblasts contains two SOX9 interaction peaks. Similarly the genes for *Osx* and *Runx2*, transcription factors required for osteoblast differentiation, contain distinct SOX9 interaction sites. One possible hypothesis is that at these sites in chondrocytes SOX9 might be part of repressor complexes. Table 3 lists the genes in which no SOX9 interaction sites were identified. This list includes the *MyoD* and *Myogenin* genes, which are master transcription factors for myoblast differentiation and are not expressed in chondrocytes. Other genes in this group include *MMP13*, those for several small leucine-rich proteoglycans, also the *Wnt 3a*, *5a*, *7a*, *9*, *Ihh* and *Shh* genes. One surprise is that no clear SOX9-interaction site was found in the *Sox6* gene, which together with *Sox5*, is required for overt chondrocyte differentiation and requires SOX9 for expression [8].

**Table 2:** Genes which contain ChIP hybridization peaks.

Extracellular Matrix Proteins					Transcription Factors					Signaling Molecules				
	Number of peaks	Promoter	Intron	3′un-translated segment		Number of peaks	Promoter	Intron	3′un-translated segment		Number of peaks	Promoter	Intron	3′un-translated segment
Col2a1	2		1 and 6		Sox9	2		1 and 2		Grb10	1		1	
Col9a2	3		1 and 4		Sox5	4	v	1 and 2		Patched	3	v	13	
Col11a1	3	v	1		Lef1	1		3		IGF2R	1		10	
Col11a2	1	v	1		Stat1	1		4		VEGF	1		1	
Col1a1	2		1	v	Tip60	1	v	1		CTGF	1	v		
Matrilin 1	3	v	3		Osx	1	v			EGFR	1		5	
Matrilin 4	4		1 and 7	v	Runx2	2		1 and 2		α-catenin	2		1 and 11	
Aggrecan	2	v												
Syndecan-3	2		1											
Cdrap	1	v	1											
ADAM-TS5	1		1											
MMP9	3		1 and 10	v						Peaks using Cut off <1.5				
Biglycan	2	v	1							BMP4	1			v
Fibromodulin	1	v	1							Chordin	1			v
Prelp	2	v								Noggin	1	v		
Chondroadherin	1		1							PTHrP	1			
										TGFβ3	2	v		
Peaks using Cut off <1.5										IL-1a	1			v
Fibronectin	1	v	1							ERK1	1		1	
Linkprotein	1		1							ERK2	1	v	1	
Epiphycan	2	v	1							PKA	1			v
Cathepsin B	1	v								p38	1		1	

**Table 3:** Genes which do not contain ChIP hybridization peaks.

Extracellular Matrix Proteins			Transcription Factors		Signaling Molecules			
Col1a2	Col3a1	MMP13	Sox6	TCF4	TGFβ1	EGF	BMP2	IGF1
Cthrc1	Lubricin	Matrilin 3	MyoD	Myogenin	TNFa	FGFR3	Integrin α11	Arhj
Osteoadherin	Osteoglycin	Asporin	β-catenin	PGC-α	Shh	Wnt3a	Wnt5a	Wnt7a
Lumican	Opticin	Decorin			Wnt9	NCAM	CD44	Asb4

### SOX9 binding to specific sequences in peaks of hybridization

**Figure 2:**
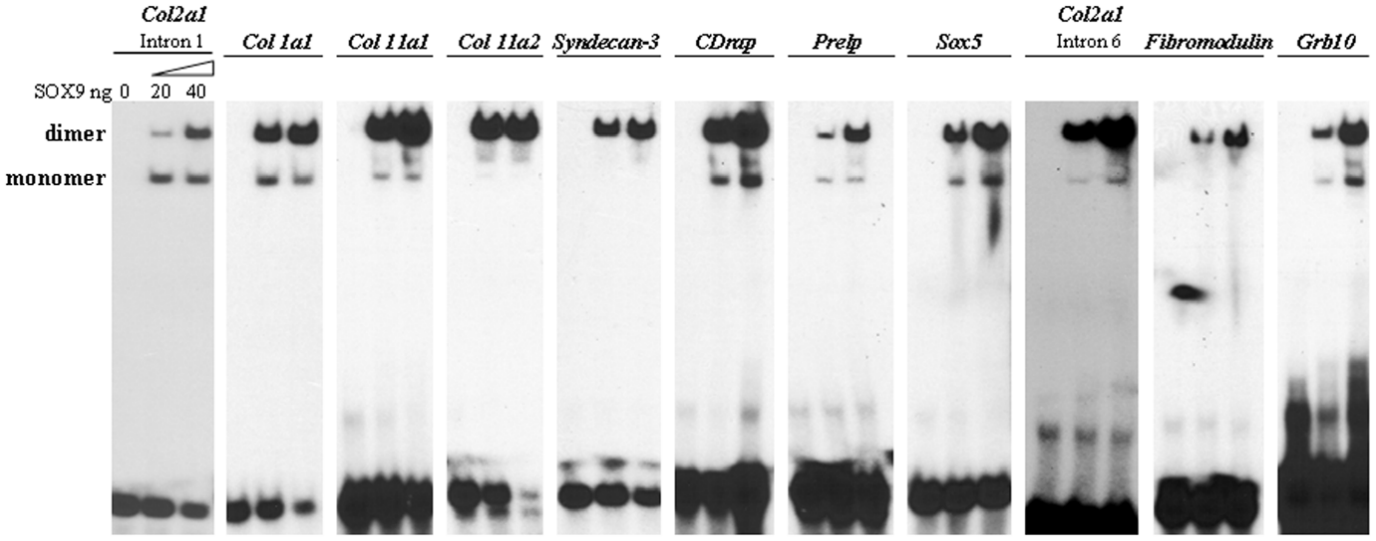
Validation of SOX9 binding motifs by EMSA. EMSA was performed as described in Materials and Methods using SOX9 binding motifs found at or near the center of the hybridization peaks in each gene. Suppl. Table S1 lists the sequences of the probes used. The mobility of ^32^P labeled probe bound to purified SOX9 was consistent with a complex containing mainly a SOX9 dimer. The amount of SOX9 used in each panel was 0, 20 or 40 ng.

We identified potential dimeric SOX9 binding sequences in peaks and verified SOX9 binding by electrophoretic mobility shift assay (EMSA) for 11 of these sites (Figure 2). These eleven sites included the sites that had been already confirmed to be SOX9 binding sites. They included sites in intron 1 of *Col2a1*, *Col11a1*, *Col11a2* and *Cdrap*. The reason we chose these sites is that by analyzing the sites by EMSA, we could validate that the ChIP-on-chip peaks revealed true SOX9 binding sites. We chose the other sites based on potential SOX9 binding sequences within the peaks of hybridization. The potential SOX9 binding sites in most of these genes diverged from the consensus binding sites, WWCAAWG(N)nCWTTGWW (W is A or T, N is non-specific base and n shows number of N) and SOX9 was indeed bound to each of these sequences. By comparing the mobility of SOX9-DNA complexes with that of a binding site in an enhancer in intron 1 of *Col2a1*, we concluded that SOX9 was mainly binding as a dimer to each of the peak sequences. We also asked whether the species conservation of sequences and the AT or GC content in the 610 bp centered on the hybridization peaks were different from 610 bp sequences surrounding random potential SOX9 binding sites outside the peaks. The conservation scores were higher in peaks than in non-peak DNA sequences (Figure S2). The AT content in the peaks was significantly lower than that in the non-peak regions indicating that SOX9 preferentially interacts with its binding sites when it is surrounded by sequences with a higher GC content (Figure S3).

### SOX9 binds to intron 6 of *Col2a1*

In addition to the hybridization peak centered on a previously identified chondrocyte-specific enhancer in intron 1 of *Col2a1*, a peak was identified in intron 6 (Figure 1, left). To validate this result we performed real-time quantitative PCR with intron 6 probes centered on the middle of the intron 6 peak using ChIP DNA generated with SOX9 antibodies. The results showed a strong signal for this DNA segment that scored higher than the DNA segment in intron 1, whereas a control segment at the 3′ end of the *Col2a1* gene gave no signal (Figure S4). A potential SOX9 dimeric binding site was identified in the PCR amplified segment and EMSA confirmed that recombinant SOX9 did indeed bind to this DNA segment as a dimer (see Figure 2). We concluded that in chondrocytes, SOX9 interacts with a specific site in the chromatin of intron 6 of the *Col2a1*.

### Intron 6 segment is likely to contribute transcriptional activity

**Figure 3:**
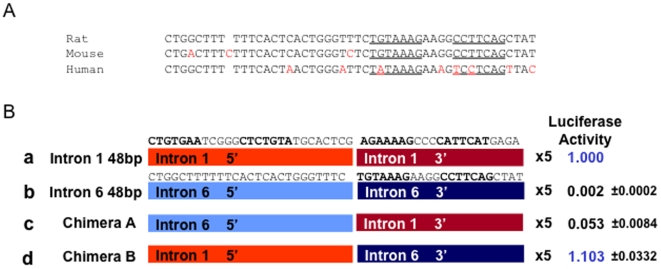
Functional analysis of SOX9 interaction site in intron 6. **A**: The 48 bp intron6 (+6676 to +6724) of rat was aligned with the corresponding sequences of mouse and human *Col2a1* gene. The putative SOX9 binding sites were underlined. **B**: **Left**; Schematic representation of chimera constructs. The sequences of a 48 bp segment in intron 1 and intron 6 are shown above each construct in a and b. The bases written by bold letters indicated the putative SOX9 binding sites. The 5′ region in intron 1 contained dimeric direct repeat, and 3′ regions of both intron 1 and intron 6 contained inverted repeats. **Right**; Each chimera construct consisted of five tandem repeats followed by a *Col2a1* minimal promoter (89bp) [73] and the *Firefly luciferase* gene and transfected in RCS cells. The values of luciferase activity were normalized by adjusting the activity of construct (a) as 1.00.

A multimerized 48 bp segment in intron 1 of *Col2a1* was previously shown to have strong SOX9-dependent enhancer activity [9]. A similar reporter construct was generated using a 48-bp sequence in the intron 6 segment that binds SOX9 in EMSA. However, the multimerized segment of intron 6 did not show SOX9-dependent enhancer activity (Figure S5 A and B). When the two multimerized sequences were placed in tandem in the reporter construct, the intron 6 sequence did not significantly increase or decrease the SOX9-dependent activity of the intron 1 enhancer in either 293T cells or RCS cells (Figure S5 A and B). The 3′ part of the 48-bp sequence in intron 1 contains a dimeric inverted repeat SOX9 binding site, whereas the 5′ part consists of a direct repeat of monomeric SOX9 binding sites (Figure 3A). When the rat intron 6 sequence was aligned with the corresponding mouse and human sequences, the mouse sequence in the 3′ region of the 48 bp was completely conserved. The human sequence in this region was not identical to rat or mouse sequences. However, the human sequence binds SOX9 efficiently in EMSA (Figure S6). Then, to test whether the inverted repeat SOX9 binding site in intron 6 could replace the inverted repeat site in the 3′ part of the 48-bp intron 1 sequence, two chimeric 48-bp sequence segments were generated as illustrated in Figure 3 and multimerized as in the original intron 1 and intron 6 vectors. When the inverted repeat SOX9 binding site of intron 1 was replaced with that of intron 6 (Chimera B), the resultant activity became similar to that of the intron 1 48-bp in RCS cells. Replacement of the 5′ sequence of the intron 1 48-bp segment with the 5′ sequence of the intron 6 48-bp (Chimera A) reduced the activity of the enhancer almost 20-fold in RCS cells. These results strongly suggested that the 5′ part of the intron 1 sequence has an important role in the Chimera B activity. In the presence of this sequence, the inverted repeat SOX9 binding site in the intron 6 segment is likely to contribute transcriptional activity in RCS cells (Figure 3B). Further when this inverted repeat sequence was mutated, SOX9 binding was abolished and the mutated Chimera B did not show any activity (Figure S7).

### Increase in intron 1 enhancer activity in the presence of intron 6

**Figure 4:**
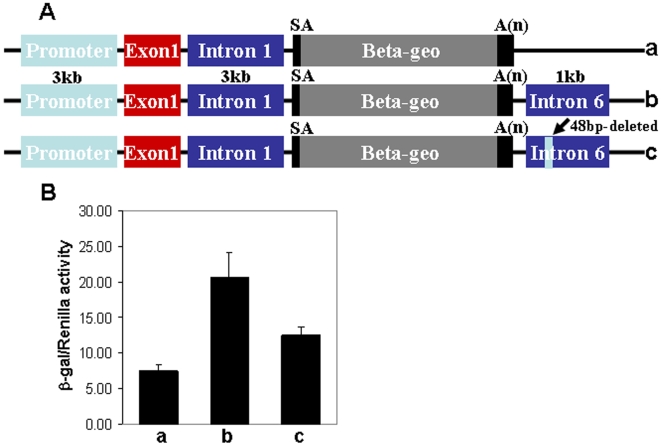
Stimulation of enhancer activity of intron 1 by intron 6. **A**: Schematic representation of constructs used. Construct (a) had a 3 kb promoter, exon 1, a 3 kb intron 1 of *Col2a1*followed by β-geo as previously shown [73]. Construct (b) had 1kb corresponding to the entire intron 6 itself (+6489 to +7374) in addition to construct (a) just after the polA. In construct (c) the SOX9 binding sequence (48bp, +6676 to +6724) was deleted from intron 6. **B**: Each construct (500 ng) was co-transfected into RCS cells with 10 ng of a TK-*Renilla* luciferase plasmid that served as an internal control for transfection efficiency. The bars represent β-galactosidase activity of each construct normalized to the internal control (TK-*Renilla luciferase* plasmid).

Although a short amplified segment of intron 6 was inactive, this DNA segment appeared to become active when juxtaposed to the 5′ segment of the intron 1 fragment. The construct containing a 3kb *Col2a1* promoter, exon 1 with a mutation in the ATG translation initiation site and 3kb of intron 1 followed by the β-geo reporter (construct a in Figure 4) has been shown to display strong chondrocyte-specific β-galactosidase expression in transgenic mice [63]. We then inserted the entire 1kb intron 6 sequence (construct b in Figure 4) or an intron 6 from which the SOX9 binding motif was deleted (construct c in Figure 4) into 3′ of the β-geo polyA signal. In transfection experiments the intron 6-containing reporter (construct b in Figure 4) was three times more active than the vector containing no intron 6. When the SOX9 motif was deleted from intron 6, the activity was decreased by about 40%. The results of these experiments suggested that the SOX9 binding motif in intron 6 might act as an enhancer and have a role in activation of the *Col2a1* gene. However, the precise role of this segment in the regulation of *Col2a1* gene in vivo remains to be clarified.

### Histone-poor SOX9 binding segment in intron 6 in chondrocytes

**Figure 5:**
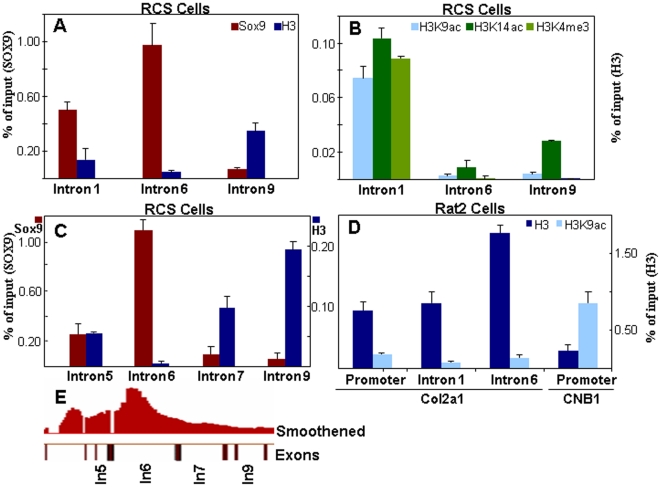
Depletion of histone H3 in intron 6 in RCS cells but not in Rat-2 fibroblast. Histone modification status in the chromatin at several introns of *Col2a1* in RCS cells (A–C) and at the promoter of *Col2a1* and *Cyclin B1* (CNB1) and introns 1 and 6 of *Col2a1* in Rat-2 fibroblasts (D) was examined by real time quantitative PCR. The ChIP DNA fragments were prepared as described in Materials and Methods using SOX9, H3 (A and C), H3K9ac, H3K14ac, H3K4me3 (B) and H3, H3K9ac (D) antibodies. The SOX9 hybridization peak in introns together with the partial map of the *Col2a1* gene is shown in E.

Judging from the experiments shown in Figure 4, the function of the SOX9 binding motif in intron 6 could be different from that in intron 1 in the *Col2a1* gene. Since histone modifications control chromatin's function in the regulation of gene expression [64], we compared the status of several histone H3 modifications around the SOX9 binding sites in intron 6 with those in other introns. These experiments revealed that the levels of histone H3 in intron 6 were clearly lower than those in intron 1 and much lower than those in intron 9, a segment with which SOX9 is not interacting (Figure 5A). The levels of H3K14ac, a marker of active chromatin, were also much reduced in intron 6 compared to their much higher levels in introns 1 and 9 (Figure 5B). This finding was in agreement with the low level of histone H3 in intron 6 (Figure 5A). As expected the levels of H3K9ac and H3K4me3 were high in intron 1 but low in introns 6 and 9, because the levels of both markers are high at the 5′ end of active genes, but decrease toward the 3′ segments (Figure 5B). When the occupancies of SOX9 and histone H3 were compared in the *Col2a1* segments immediately surrounding intron 6, the high occupancy of SOX9, and the very low occupancy of histone H3 were restricted to intron 6 (Figure 5C). Overall these results strongly suggest that the chromatin segment surrounding the SOX9 binding site in intron 6 was depleted of nucleosomes. We then asked whether the absence of histone H3 in intron 6 was also found in the chromatin of a cell type that does not express *Col2a1*. Figure 5D shows that histone H3 occupancy in intron 6 of *Col2a1* in Rat-2 fibroblasts, in which C*ol2a1* is not expressed, was much higher than in RCS cells. The occupancy of the active gene marker, H3K9ac was low in the different segments of *Col2a1* in Rat-2 fibroblast, compared to its higher occupancy in the promoter region of the *cyclin B1* gene, which is active in Rat-2 fibroblast. Further we performed Chip-qPCR with SOX9 and H3 antibodies using two additional cell types, one consists of mouse primary rib chondrocytes that express *Col2a1* and *Sox9* and the other is a human lymphoblast cell line (Reh) that expresses neither *Col2a1* nor *Sox9* (Figure S8). Our results support the hypothesis that the histone-poor region of intron 6 is specific for the cells that express *Col2a1*.

## Discussion

Our objective was to identify possible SOX9 chromatin interaction sites among a number of genes, which are believed to be involved either in chondrocyte differentiation or in chondrocyte function. The 80 genes, which were placed on a high density oligonucleotide array, were subdivided in three groups, namely genes for extra cellular matrix components, for transcription factors, and for signaling molecules. As chromatin source for ChIP-on-chip experiments, we used rat chondrosarcoma cells (RCS cells), because these cells display many chondrogenic characteristics including secretion of specific cartilage ECM proteins and high contents of SOX9, SOX5 and SOX6 (see Figure S1) [19]. In arrays containing either sense or anti-sense oligonucleotides the SOX9 interaction profiles showed very similar patterns of hybridization providing strong evidence for the high degree of specificity of these hybridization peaks. Further, the profile of the negative controls using non-specific IgGs showed essentially no hybridization peaks. Overall these results showed that the ChIP-on-chip approach was very efficient in identifying highly specific SOX9 interaction sites. Among the 80 genes placed on the array, 30 genes showed one or more clear hybridization peaks. Most of these genes are active during chondrocyte differentiation and include extracellular matrix (ECM) protein-coding genes, genes for transcription factors and for signaling proteins. Our experiments also indicate that among the ECM gene several genes for small leucine rich proteoglycans show clear interaction sites for SOX9. Previous experiments identified functional SOX9 binding sites that control the activity of chondrocyte-specific transcriptional enhancers in the *Col2a1*, *Col11a2* [10], *Aggrecan* [12] and *Cdrap* [11] genes. Our finding that the SOX9 hybridization peaks are centered on these previously identified SOX9 binding sites strongly supports the hypothesis that the SOX9 interaction sites have a clear biological role. The results of our ChIP-on-chip experiments revealed that most cartilage ECM genes contain at least two SOX9 chromatin interaction sites. These sites are mainly located in promoter regions and in first introns, some also in other introns, and fewer in 3′ un-transcribed regions. Since promoter and first intron segments contain major transcriptional regulatory sequences the presence of SOX9 interaction sites in these DNA segments suggests that in these genes SOX9 may interact with the general transcriptional machinery.

Our previous results have shown that in the absence of SOX9 there is no expression of *Sox5* and *Sox6* in chondrocytes [8]. *Sox5* contains several SOX9 interaction sites located in the promoter, introns 1 and 2. In contrast, we did not detect SOX9 interaction sites in the *Sox6* gene. This suggests either that the *Sox6* gene is not a direct target of SOX9 or that the SOX9 interaction sites are located outside the *Sox6* DNA segments that were printed on the array. Our experiments detected interaction sites for SOX9 in the *Sox9* gene located in introns 1 and 2. Although our previous results have indicated that chondrocyte-specific regulatory segments of *Sox9* were still active even when the *Sox9* gene was partially deleted, it is nevertheless possible that the SOX9 binding sites in introns 1 and 2 have an autoregulatory role in the transcriptional control of SOX9 in combination with other transcription factors. In Sertoli cells SOX9 binds to the promoter region of *Sox9* but our results did not detect a SOX9 interaction sites in the chromatin of this segment in chondrocytes [65]. The PTHrP receptor is highly expressed in prehypertrophic chondrocytes where *Sox9* is equally highly expressed. Through this receptor and via PKA, PTHrP stimulates the phosphorylation of SOX9 [17], [54]. This phosphorylation of SOX9 increases its activity [17]. The finding of a SOX9 interaction site in the gene for the PTHrP receptor indicates that SOX9 interacts with the gene for a component of a signaling pathway that increases the activity of SOX9. BMP signaling also plays a major role in chondrocyte differentiation. Finding SOX9 interaction sites in genes of the BMP signaling strongly suggested that SOX9 is directly implicated in the control of these genes. Interestingly, the *Bmp4* gene itself showed also a SOX9 interaction site. In addition to the genes specifically expressed in chondrocytes such as cartilage ECM genes, SOX9 interaction sites were also detected in ubiquitously expressed genes such as those for the transcription factors Tip60, Stat1 and Lef1, and for the ERK1, ERK2 and PKA signaling molecules. Thus in chondrocytes SOX9 also appeared to interact with a number of genes that are more broadly expressed. For example, we showed recently that Tip60 up-regulated expression of *Col2a1*, a direct target of SOX9 [31]. Our present data indicated that SOX9 interacted with the *Tip60* promoter and up-regulated this promoter (H.Yasuda et al., unpublished results). Thus SOX9 interacts with the gene for a coactivator, which cooperates with SOX9 in activating a downstream target of SOX9. Previous experiments have shown that both ERK1 and ERK2 signaling in response to FGF increases SOX9 expression [15], whereas signaling by p38 also in response to FGF increases SOX9 activity [66]. Interestingly, the well known *Col1a1* gene, which is not expressed in chondrocytes and in RCS cells, showed two clear interaction sites one in intron1, the other immediately 3′ to the gene. One possible hypothesis is that SOX9, which is known to be a transcriptional activator, might also be able to become part of a negative transcriptional complex and that in chondrocytes SOX9 may have a role in silencing genes that are active in *Sox9*-expressing osteochondroprogenitor cells as well as in osteoblasts. The SOX9 interaction sites in the genes for RUNX2 [32] and OSX [33], two transcription factors that are required for osteoblast differentiation, may have a similar function. The genes for two other “master” transcription factors, MyoD [34] and Myogenin [35] which are not expressed in chondrocytes but are needed for myoblast differentiation, did not have SOX9 interaction sites consistent with the high degree of specificity of the role of SOX9 in the chromatin of chondrocytes.

EMSA of a sample of SOX9 interaction sites confirmed that SOX9 was able to bind to specific sites found in the hybridization peaks of our ChIP-on-chip experiment. These sequences often diverged from the consensus dimeric binding sites WWCAAWGX(N)CWTTGWW (W = A or T) by several mismatches. Among the many genes having SOX9 binding sites, the *Col2a1* gene is one that has been most intensively characterized so far in terms of response to SOX9. In this gene, SOX9 has been shown to bind to a sequence in a chondrocyte-specific enhancer in intron 1 and consequently to induce the activity of this enhancer. As shown here a ChIP-on-chip peak was clearly centered on this binding site in intron 1. In addition to this peak, another peak was detected in intron 6 of *Col2a1*. The binding of SOX9 at this site was validated by EMSA and ChIP-qPCR. Intron 6 increases the activity of a reporter containing the *Col2a1* promoter and the enhancer of intron1, and the deletion of a short segment containing the SOX9 binding site in intron 6 from this reporter decreased its activity suggesting that this SOX9 binding site functions as a positive regulatory site in *Col2a1*. The 3′ sequence of a 48bp of intron 6 contained an inverted repeat sequence similar to the inverted repeat sequence in the 3′segment of the 48bp in intron 1 (Figure 3). The 3′ segment of intron 6 was able to functionally substitute for the inverted repeat sequence in intron 1 when tested as a chimeric construct containing the 5′ part of the 48 bp of intron1. We previously showed that a highly multimerized repeat of an 18 bp sequence containing the inverted repeat in intron 1 was sufficient for enhancer activity in chondrocytes [67]. A similar construction containing the inverted repeat of intron 6 was not tested in this study. However, the function of this region in the regulation of the *Col2a1* gene in vivo still remains to be clarified.

Very interestingly the intron 6 enhancer segment is either very poor in nucleosomes or free of nucleosomes in chondrocytic cells (RCS cells and mouse primary rib chondrocyte cells [68]) (Figure 5 and S8). Nucleosome deficient regions in the genome have been detected in promoter regions of actively transcribed genes, in 3′ non-translated segments and in interval sequences between genes [69]–[71]. Recently nucleosome-deficient structures were also shown at several transcription factor binding sites in *Saccharomyces cerevisiae* [72], [73]. Although the DNA sequence itself plays an important role in nucleosome occupancy, it is also likely that competition between the binding of transcription factors to their recognition sites and of nucleosomes determines their relative occupancy at specific sites in the genome. Since intron 6 in the Rat-2 fibroblasts and human lymphoblast, Reh cells, in which both *Col2a1* and *Sox9* genes were not actively transcribed, did form nucleosome structures (Figure 5 and S8), we propose that SOX9 is part of a large multiprotein complex that occupies the chromatin in intron 6 in chondrocytes and prevents nucleosome structures to form. Based on our transfection experiments it is likely that this complex, together with a SOX9-containing complex that occupies the enhancer segment in intron 1, interacts with the pre-initiation complex at the promoter to activate the *Col2a1* gene in chondrocytes. One possible explanation for this nucleosome free structure is that several transcription factors are recruited, disrupt the nucleosome structure and bind to this region through the binding to SOX9. In summary, the ChIP-on-chip experiment using chondrocyte chromatin has identified SOX9 interaction sites in a number of genes for components of cartilage as well as for transcription factors and signaling molecules that participate in the regulation of the chondrocyte program. Our experiments also indicate that SOX9 co-opts other genes that are largely ubiquitous to become part of the SOX9 program in chondrocytes. Because SOX9 interacts also with genes that are not expressed in chondrocytes but are expressed in a cell type that is derived from a common progenitor, SOX9 could have a negative role in these genes. Other experiments led to the identification of a nucleosome-free novel SOX9-dependent segment in the *Col2a1* gene. Overall our experiments are providing new insights in the essential role of SOX9 in the complexity of the chondrocyte genetic program.

## Materials and Methods

### Cell culture

Human HEK293T, Reh cells and Rat fibroblast (Rat-2) were obtained from American Type Culture Collection (ATCC). Rat chondrosarcoma (RCS) cells were gifted from Dr. James H. Kimura, Henry Ford Hospital, Detroit, Michigan [19]. HEK293T, Rat-2, and RCS cells were cultured in DMEM and Reh cells were in RPMI1640 supplemented with 10% fetal bovine serum.

### ChIP-on-chip and ChIP-qPCR analysis

ChIP was performed according to the previously described method [74] using the ChIP assay kit (Millipore Co., Ltd). Briefly, the RCS cells were fixed with formaldehyde and then the chromatin prepared by sonication was treated with rabbit anti-SOX9 antibodies (Millipore, AB5809) or non-specific rabbit IgGs. The resultant DNA fragments were ligated with random oligonucleotides after the DNA was modified with terminal deoxyribonucleotide transferase (TdT). The modified anti-SOX9 precipitated and IgG precipitated DNA fragments were amplified by PCR and further labeled with Cy3 and Cy5, respectively. The chip array was done by use of NimbleGen platform (NimbleGen). The ChIP-qPCR experiments were carried out by SYBR Green PCR Master Mix and ABI7900HT (Applied Biosystems) using ChIP DNA as a template. ChIP DNA-to-input DNA ratios were calculated after immunoprecipitation with each antibody. The data were normalized with IgG control antibody, The primers used for the qPCR are shown in Suppl. Table S2.

### Electrophoretic mobility shift assay

The probes used in EMSA shown in Suppl. Table S1 were labeled by α^32^P-dCTP using Klenow fragment, and then EMSA was performed using recombinant human SOX9 proteins expressed in *E.coli* as described previously [67].

### Plasmid construction and reporter assay

Oligonucleotides of 48 base pair fragments of both *Col2a1* intron 1 and intron 6 were cloned into pBluescript vector and then multimerized as previously described [67]. For the luciferase reporter assay we used a luc4 reporter plasmid. The intron 6 DNA was obtained by PCR using Bac CH230-103H12 provided from BACPAC Resource Center (Oakland, USA) as a template. In the reporter assay, the cells were co-transfected with an adequate reporter plasmid, a SOX9 expressing plasmid and a control plasmid (TK-*Renilla* luciferase plasmid) using Fugene 6, The luciferase and β-galactosidase activities were obtained by use of a dual luciferase assay system (Promega Co. Ltd) and a Tropix Galacto Reaction kit (Applied Biosystems), respectively. Each value in the reporter assay was presented as the fold increase in *Firefly* luciferase activity units or β-galactosidase activity units per *Renilla* luciferase activity units from three independent experiments, each performed in triplicate.

## Supporting Information

Table S1 List of Primers for EMSA
(0.05 MB DOC)

Table S2 List of Primers for qPCR
(0.08 MB DOC)

Table S3 List of Primers for Figure S1
(0.05 MB DOC)

Figure S1 mRNA expression levels in RCS cells compared to Rat-2 fibroblast cells. Total RNA was extracted from logarithmically growing RCS cells or Rat-2 cells using Trizol reagent (Invitrogen) according to the manufacturer's protocol. cDNA was prepared from the RNA using AMV reverse transcriptase followed by qPCR with specific primer for each RNA (Table S3) using SYBR Master Mix and ABI 7900 (Applied Biosystems). The difference of Ct values (delta Ct) between the Ct value of each sample and that of GAPDH was calculated. Then the delta Ct value of each gene in RCS cells was compared to that value in Rat-2 cells. The values on the Y axis show expression levels in RCS cells compared to Rat-2 cells as log_2_y.
(1.56 MB TIF)

Figure S2 Sequence conservation of peaks. By use of the program, Multiz9way, obtained from UCSC genome browser, the evolutionary conservation was measured in nine vertebrates including rat, human, mouse, dog, cow, opossum, chicken, frog and zebrafish. In order to calculate the conservation scores, 72 regions out of 76 peaks that contain the consensus inverted repeat, WWCAAWG(N)nCWTTGWW (W is A or T, N is non-specified base and n shows number of N.) with a space (n) of 3 to 6. These regions also conserved a core inverted repeat sequence, AANG(N)nCNTT, and had a maximum of 2 mismatches in each half of the consensus repeat. 76 non-peak regions containing such repeat were also chosen. Note that such sequences are frequently found in both peak and non-peak regions of the genome. A two-sample t-Test showed that p-value was 0.01864. Readers interested in the detailed sequences that were used to compose this figure should contact the corresponding author.
(1.56 MB TIF)

Figure S3 Box plot showing AT content of peak and non-peak regions. We compared AT or GC content in 610 bp sequences centered on the hybridization peaks were compared to 610 bp sequences surrounding random potential SOX9 binding sites outside the peaks. The bold horizontal lines show the mean of the data. Mean of AT content in peak regions was 46.7%, and mean AT content in non-peak regions was 53.8%. By Student's t-Test, p-value was measured at 3.425×10^−9^.
(1.56 MB TIF)

Figure S4 Validation of SOX9 binding sites in *Col2a1* on ChIP-on-chip microarray by ChIP-qPCR. The DNA obtained from ChIP of sheared chromatin of RCS cells with SOX9 antibodies was used as the template in real time qPCR to amplify a segment of intron 6 of *Col2a1*. A segment of intron 1 of *Col2a1* previously identified as containing a functional SOX9 binding sites and another segment located 3′ to the *Col2a1* gene served as positive and negative controls, respectively. Error bars represent standard deviations. The sequence of each probe is shown in Table S2.
(1.56 MB TIF)

Figure S5 Functional analysis of the SOX9 binding site in intron 6. Five tandem repeats of the 48bp in intron1, the 48bp in intron6 or the segment conjugated each other were inserted in 5′ to the *Col2a1* minimal promoter (89bp) followed by the firefly luciferase gene (Luc4) [67]. The activity of each construct was tested by measuring the activity of each reporter in 293T (A) and RCS cells (B). 293T cells were transiently transfected with the reporter plasmids in the presence or absence of 0.5 µg of SOX9 expression plasmid, whereas RCS cells were transfected only with the reporter. Five tandem repeats of a 48 bp sequence in intron 1 (5xIn1) showed strong enhancer activity, but five tandem repeats of an equivalent 48 bp in intron 6 (5xIn6) showed no transcriptional activation. The duplication of this construct (5xIn6, 5xIn6) did not show activity in either cell. However, the combination of the intron 1 and intron 6 sequence (5xIn6, 5xIn1) did not repress intron 1 enhancer activity in both cells and rather increased slightly the activity in 293T cells (A). Each experiment included 0.5 µg of the reporter plasmid and 0.01 µg of an internal control plasmid, TK-*Renilla* luciferase construct, to normalize for transfection efficiency.
(1.56 MB TIF)

Figure S6 Conservation of the SOX9 binding site in intron 6 among different species. The sequences of Sox9 binding sites in intron 6 of *Col2a1* gene of three different species are aligned. The inverted repeat of the rat SOX9 binding site is underlined. The bases in red are the bases that are not identical to the corresponding human sequence. The sequence of the binding sites between rat and mouse are completely conserved. The sequence of the binding site of the human is not identical to the rat sequence. B. An EMSA assay was performed to test whether SOX9 was binding to the human intron 6 sequence. The sequence of each probe is shown in Table S3. The human putative SOX9 binding site binds SOX9 efficiently.
(1.56 MB TIF)

Figure S7 Effect of mutation on the enhancer activity of Chimera B shown in figure 3. The seven bases of 3′ region of the 48bp of intron 6 were mutated as follows. The bases mutated are shown by italics. Wild: CTGGGTTTCTGTAAAGAAGGCCTTCAGCTATCTGA Mutant; CTGGGTTTCTGT*CG*A*A*AAGG*AAAA*CAGCTATCTGA The ability of this mutated fragment to bind Sox9 was demonstrated as shown in Figure 2. Lane 1; Control probe (SOX9 binding site of *Col2a1* intron 1), lane 2; SOX9 binding site of *Col2a1* intron 6, lane 3; mutant SOX9 binding site of *Col2a1* intron 6. B. Luciferase reporter assay of Chimera B and mutant Chimera B constructs. By use of this mutant fragment, the mutant Chimera B (Figure 3) construct was prepared and its enhancer activity was compared with wild Chimera B construct using RCS cells. Reporter assay was done as shown Figure 3. The mutant Chimera B did not show the enhancer activity.
(1.56 MB TIF)

Figure S8 Depletion of histone H3 in 2^nd^ peak of *Col2a1* gene in primary chondrocyte. Binding of H3 and Sox9 in *Col2a1* gene of mouse rib chondrocyte primary culture and human lymphoblast, Reh cells, was demonstrated by ChIP-qPCR The mouse chondrocytes were cultured as shown previously [68]. The ChIP-qPCR was performed as shown in Figure 4. The primers used in this figure are shown in Table S2. 1^st^ peak and 2^nd^ peak correspond to the peaks in intron 1 and intron 6 of rat *Col2a1* gene, respectively. Expression of *Col2a1* and *Sox9* was detected in primary chondrocyte cells but not in Reh cells by RT-qPCR method shown in Figure S1. The sequence including SOX9 binding site corresponding to intron 6 of the mouse and rat *Col2a1* gene is highly conserved in the human *Col2a1* gene.
(1.56 MB TIF)
