# Supplementary material for: Identification of SOX9 Interaction Sites in the Genome of Chondrocytes
Source: PLoS One. 2010 Apr 9;5(4):e10113. doi: 10.1371/journal.pone.0010113 (PMC2852419; doi:10.1371/journal.pone.0010113)
Supplement: Table S1 — List of Primers for EMSA (0.05 MB DOC) [file pone.0010113.s001.doc]

Table S1. List of Primers for EMSA

| EMSA probe | |
| --- | --- |
| Col2a1 intron1 (mouse) | ggcgcttgagaaaagccccattcatgagagg |
| Col2a1 intron1 (rat) | ggcactcgagaaaagccccattcatgagagg |
| Col1a1 (rat) | gggggggacaacaaagagagcattgtatcacactct |
| Col11a1 (rat) | ggattttgcctgaaagaaccCtTTaacaagcaatga |
| Col11a2 (rat) | ggccctcccgacaaagcacgccttgtatcccccacc |
| Syndecan-3 (rat) | ggtggatcaaaccaaagcagccttctcctccctcaa |
| Cdrap (rat) | gggtctgggaacaaggcagttctttgatttgtggtg |
| Matrilin4 (rat) | ggctgagacaggaatgaatcactttcctgggggtgg |
| Prelp (rat) | gggatgcccgctaacggctcacttttaatgtttcct |
| Sox5 (rat) | ggttatgaggataaaggagccttgatgggaaaaagt |
| Col2a1 intron6 (rat) | ggcagatagctgaaggccttctttacagaaacccag |
| Col2a1 intron6 (human) | ggcaggtaactgaggactttctttatagaatcccag |
| Col2a1 intron6 (human, mutant) | ggcagatagctgttttccttttcgacagaaacccag |
| Fibromodulin (rat) | ggagcctcacagaaagctgagctttgaagaggaagg |
| Grb10 (rat) | ggacccaaaactaatgttgcacattttactgccttc |

The gg in the 5’ of each sequence was added for the labeling by ^32^P-dCTP using Klenow fragment. The bases written by capital letter showed the proposed core motif of SOX9 binding sequence. The location of each sequence in each gene is obtained from Ensembl Gene predictions and shown below.

*Col2a1* intron 1 (mouse)(Chr 15:97832837-97832865), *Col2a1* intron 1 (rat) (Chr 7: 136701250-136701282), *Col1a1*(Chr 10: 83603468-83603501), *Col11a1* (Chr 2: 209999224-209999257), *Col11a2* (Chr 20: 4954163-4954196), *Syndecan-3* (Chr 5: 149516776-149516809), *Cdrap* (Chr 1: 82257499-82257532), Matn4 (Chr 3: 155415696-155415729), *Prelp* (Chr 13: 46874413-46874446), *Sox-5* (Chr 4: 181820328-181820361), *Col2a1* intron6 (rat) (Chr 7: 136701250-136701282), *Col2a1* intron6 (human) (Chr 12: 48391149-48391182), *Fibromodulin* (Chr 13: 46986880-46986913), *Grb10* (Chr 14: 92842027-92842060).
