# Supplementary material for: Identification of SOX9 Interaction Sites in the Genome of Chondrocytes
Source: PLoS One. 2010 Apr 9;5(4):e10113. doi: 10.1371/journal.pone.0010113 (PMC2852419; doi:10.1371/journal.pone.0010113)
Supplement: Table S2 — List of Primers for qPCR (0.08 MB DOC) [file pone.0010113.s002.doc]

Table S2. List of Primers for qPCR

| The Primers for qPCR | | |
| --- | --- | --- |
| *Col2a1* gene | | |
| Promoter | FW | TGGGTACCTTTGTTAGCCATGA |
|  | RV | CTGAAAAGAACGGGAAAAGGAA |
| Intron 1 | FW | TGAAACCCTGCCCGTATTTATT |
|  | RV | GCCTTGCCTCTCATGAATGG |
| Intron 5 | FW | CACCCTTGACAGGAAGAAAAAAAG |
|  | RV | TCCTCTAAAGGCCTGGGTACCT |
| Intron 6 | FW | CCCGTCGTGCGGTTAATT |
|  | RV | ACTGCTCTTCCAGAGAAACACAAGT |
| Intron 7 | FW | CCTCATTCTTCCTAGCCCCTCTA |
|  | RV | CATTTAGCCCCAGGAGTTTGG |
| Intron 9 | FW | GCAACAGGGAGTGCCATAGG |
|  | RV | AGCTCCTGTGATATTACTTCTCTGCAA |
| 3’ end | FW | AGTGCATGGTTTCCAAGGTTCTT |
|  | RV | AACAGCTGCTACTAAATTGACCCTAGA |
| *CNB1* gene | | |
| Promoter | FW | ACCTAAAGCAGGTGGCAGCAAAGAG |
|  | RV | CAGAGGTTCGCCGATCTGAGAAG |

| Human Col2a1 Intron 1 | FW | TCCGCGAGGAACCAGTTTAA |
| --- | --- | --- |
|  | RV | TGTTTGCATTGGGAGATTGG |
| Human Col2a1 Intron 6 | FW | CGTGGTACAGATGCCAGGAA |
|  | RV | GCTAAGGGCTGGCTTTTTTCA |
| Human Col2a1 3’ | FW | AAGAGTAGGAAGAAATGGGAAGCA |
|  | RV | CCCTGGGCCTGCACTTAAA |
| Mouse Col2a1 Intron 1 | FW | TGAAACCCTGCCCGTATTTATT |
|  | RV | GCTTTTCTCAAGCGCATACAGA |
| Mouse Col2a1 Intron 6 | FW | TCTTTCACTCACTGGGTCTCTGTAA |
|  | RV | CCCAAGAATTAACCGCATGGT |
| Mouse Col2a1 3’ | FW | GCTCGTGACAAGCAAGGAAAT |
|  | RV | AGGGACACTCTCTACCATTGAGCTA |
