# Supplementary material for: Identification of SOX9 Interaction Sites in the Genome of Chondrocytes
Source: PLoS One. 2010 Apr 9;5(4):e10113. doi: 10.1371/journal.pone.0010113 (PMC2852419; doi:10.1371/journal.pone.0010113)
Supplement: Table S3 — List of Primers for Figure S1 (0.05 MB DOC) [file pone.0010113.s003.doc]

Table S3. List of Primers for Figure S1

| The primers for Figure S1 | | |
| --- | --- | --- |
| Rat Prelp | FW | GTGGGATGATTGGGACCTTGC |
|  | RV | TCCATCTGTCTTCCCTGACTGTCCA |
| Rat Syndecan | FW | GTGGCTGCAGTGGTAGATGTG |
|  | RV | ACAGAAGTTGTAGAAGAGCCCAG |
| Rat Matrilin 1 | FW | CGTCATTAATGTAGTCCTGGCTCC |
|  | RV | AAGTATCTCATAGACAATTCCTTCACTGTG |
| Rat Cdrap | FW | CCAGGTCTCCATAGTAATCTCCCTG |
|  | RV | ACAGGGGCCAAGTGGTATATG |
| Rat Col11a1 | FW | CCTGGAAATAACTGCTTCGTTGG |
|  | RV | AGCAGAAAGAATTCTAAAGATCCAGATATCG |
| Rat Aggrecan | FW | GGTCTTTTCAGTTCTCTCCCCAG |
|  | RV | TGCAAAGCCCATCTTCGACATG |
| Rat GAPDH | FW | CCAGTAGACTCCACGACATACTCAG |
|  | RV | GGAAACCCATCACCATCTTCCAG |
| Rat Sox6 | FW | TTGGGATCATCTTCGTAGTCGTCATAG |
|  | RV | TATGAAGATGGACGGCGCGAG |
| Rat Fibromodulin | FW | GGTTGTTGTGGTCCAGGTACAG |
|  | RV | GCAACCAGATTACCAGTGACAAGATAG |
| Rat Sox9 | FW | CATCAAGACGGAGCAACTGAG |
|  | RV | GTGGTCGGTGTAGTCATACTGC |
| Rat Col2a1 | FW | TCAACAATGGGAAGGCGTGAG |
|  | RV | GTTCACGTACACTGCCCTGAAG |
| Rat Sox5 | FW | GACACCGACTCCCCACTCTGTC |
|  | RV | CGATGGCAATAAAGTTATGTCTTCATTAGC |
| Rat Col1a1 | FW | TGTCCATTCCGAATTCCTGGTCTG |
|  | RV | CGAATACAAAACCACCAAGACCTC |
