# Supplementary figures and images for: Identification of SOX9 Interaction Sites in the Genome of Chondrocytes
Source: PLoS One. 2010 Apr 9;5(4):e10113. doi: 10.1371/journal.pone.0010113 (PMC2852419; doi:10.1371/journal.pone.0010113)

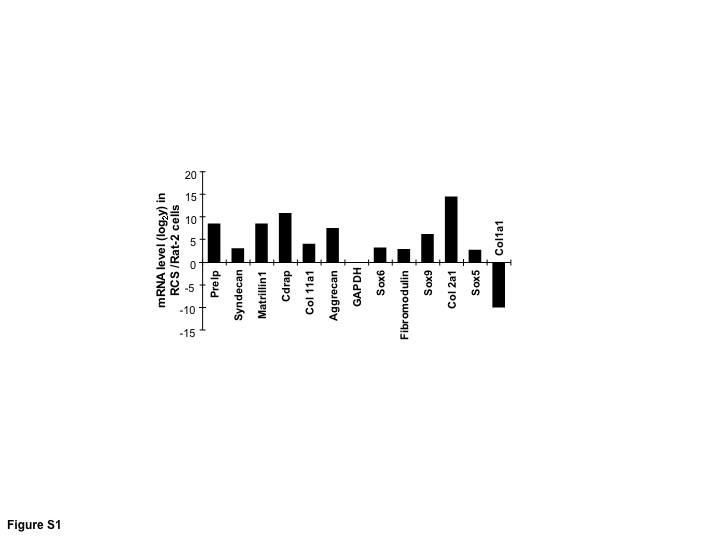

Supplement: Figure S1 — mRNA expression levels in RCS cells compared to Rat-2 fibroblast cells. Total RNA was extracted from logarithmically growing RCS cells or Rat-2 cells using Trizol reagent (Invitrogen) according to the manufacturer's protocol. cDNA was prepared from the RNA using AMV reverse transcriptase followed by qPCR with specific primer for each RNA (Table S3) using SYBR Master Mix and ABI 7900 (Applied Biosystems). The difference of Ct values (delta Ct) between the Ct value of each sample and that of GAPDH was calculated. Then the delta Ct value of each gene in RCS cells was compared to that value in Rat-2 cells. The values on the Y axis show expression levels in RCS cells compared to Rat-2 cells as log2y. (1.56 MB TIF) [file pone.0010113.s004.tif]

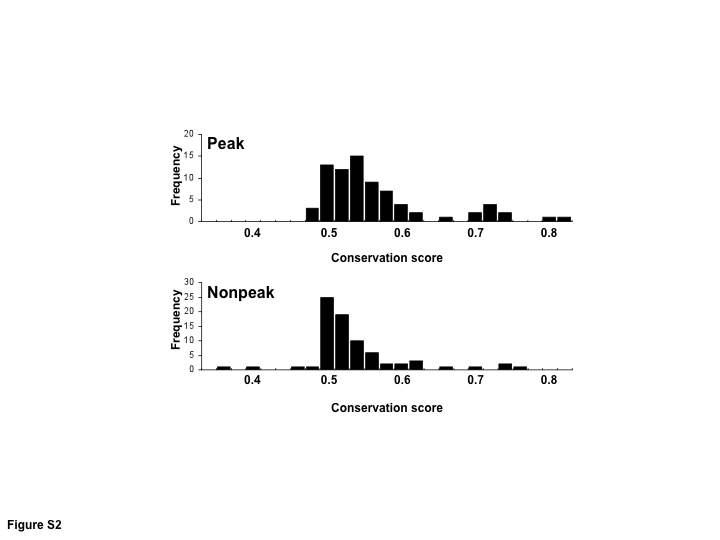

Supplement: Figure S2 — Sequence conservation of peaks. By use of the program, Multiz9way, obtained from UCSC genome browser, the evolutionary conservation was measured in nine vertebrates including rat, human, mouse, dog, cow, opossum, chicken, frog and zebrafish. In order to calculate the conservation scores, 72 regions out of 76 peaks that contain the consensus inverted repeat, WWCAAWG(N)nCWTTGWW (W is A or T, N is non-specified base and n shows number of N.) with a space (n) of 3 to 6. These regions also conserved a core inverted repeat sequence, AANG(N)nCNTT, and had a maximum of 2 mismatches in each half of the consensus repeat. 76 non-peak regions containing such repeat were also chosen. Note that such sequences are frequently found in both peak and non-peak regions of the genome. A two-sample t-Test showed that p-value was 0.01864. Readers interested in the detailed sequences that were used to compose this figure should contact the corresponding author. (1.56 MB TIF) [file pone.0010113.s005.tif]

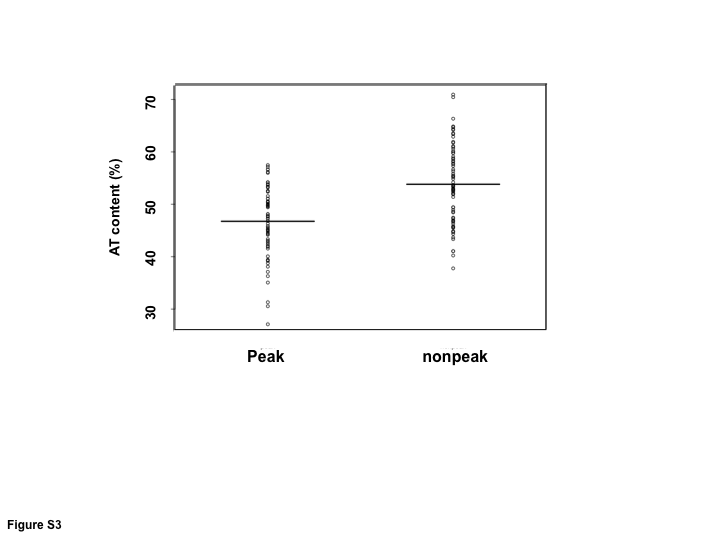

Supplement: Figure S3 — Box plot showing AT content of peak and non-peak regions. We compared AT or GC content in 610 bp sequences centered on the hybridization peaks were compared to 610 bp sequences surrounding random potential SOX9 binding sites outside the peaks. The bold horizontal lines show the mean of the data. Mean of AT content in peak regions was 46.7%, and mean AT content in non-peak regions was 53.8%. By Student's t-Test, p-value was measured at 3.425×10^−9^. (1.56 MB TIF) [file pone.0010113.s006.tif]

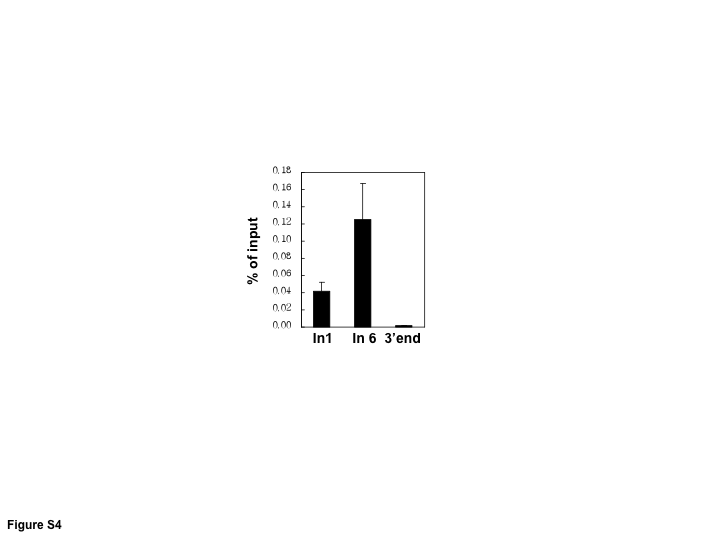

Supplement: Figure S4 — Validation of SOX9 binding sites in Col2a1 on ChIP-on-chip microarray by ChIP-qPCR. The DNA obtained from ChIP of sheared chromatin of RCS cells with SOX9 antibodies was used as the template in real time qPCR to amplify a segment of intron 6 of Col2a1. A segment of intron 1 of Col2a1 previously identified as containing a functional SOX9 binding sites and another segment located 3′ to the Col2a1 gene served as positive and negative controls, respectively. Error bars represent standard deviations. The sequence of each probe is shown in Table S2. (1.56 MB TIF) [file pone.0010113.s007.tif]

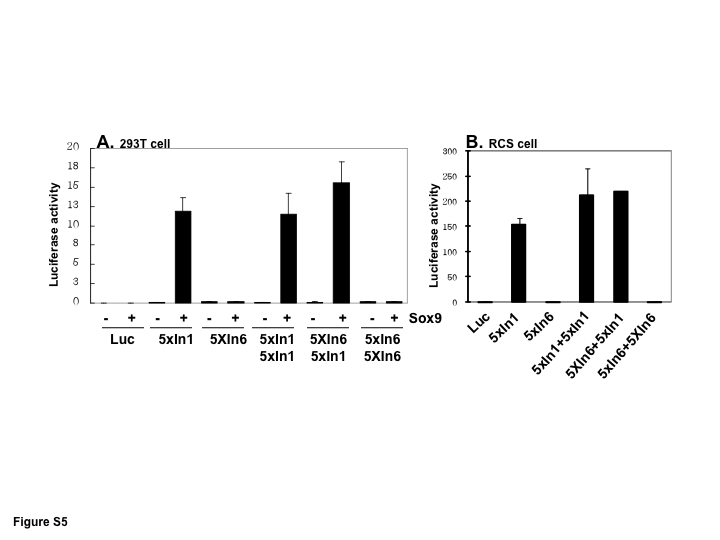

Supplement: Figure S5 — Functional analysis of the SOX9 binding site in intron 6. Five tandem repeats of the 48bp in intron1, the 48bp in intron6 or the segment conjugated each other were inserted in 5′ to the Col2a1 minimal promoter (89bp) followed by the firefly luciferase gene (Luc4) [67]. The activity of each construct was tested by measuring the activity of each reporter in 293T (A) and RCS cells (B). 293T cells were transiently transfected with the reporter plasmids in the presence or absence of 0.5 µg of SOX9 expression plasmid, whereas RCS cells were transfected only with the reporter. Five tandem repeats of a 48 bp sequence in intron 1 (5xIn1) showed strong enhancer activity, but five tandem repeats of an equivalent 48 bp in intron 6 (5xIn6) showed no transcriptional activation. The duplication of this construct (5xIn6, 5xIn6) did not show activity in either cell. However, the combination of the intron 1 and intron 6 sequence (5xIn6, 5xIn1) did not repress intron 1 enhancer activity in both cells and rather increased slightly the activity in 293T cells (A). Each experiment included 0.5 µg of the reporter plasmid and 0.01 µg of an internal control plasmid, TK-Renilla luciferase construct, to normalize for transfection efficiency. (1.56 MB TIF) [file pone.0010113.s008.tif]

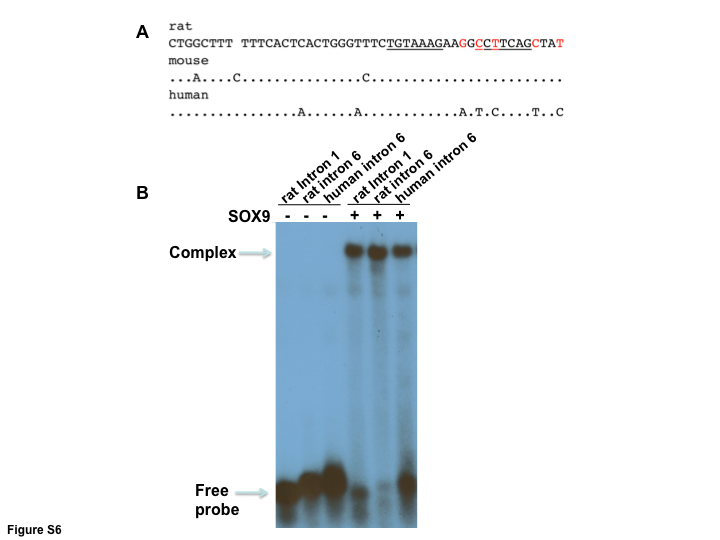

Supplement: Figure S6 — Conservation of the SOX9 binding site in intron 6 among different species. The sequences of Sox9 binding sites in intron 6 of Col2a1 gene of three different species are aligned. The inverted repeat of the rat SOX9 binding site is underlined. The bases in red are the bases that are not identical to the corresponding human sequence. The sequence of the binding sites between rat and mouse are completely conserved. The sequence of the binding site of the human is not identical to the rat sequence. B. An EMSA assay was performed to test whether SOX9 was binding to the human intron 6 sequence. The sequence of each probe is shown in Table S3. The human putative SOX9 binding site binds SOX9 efficiently. (1.56 MB TIF) [file pone.0010113.s009.tif]

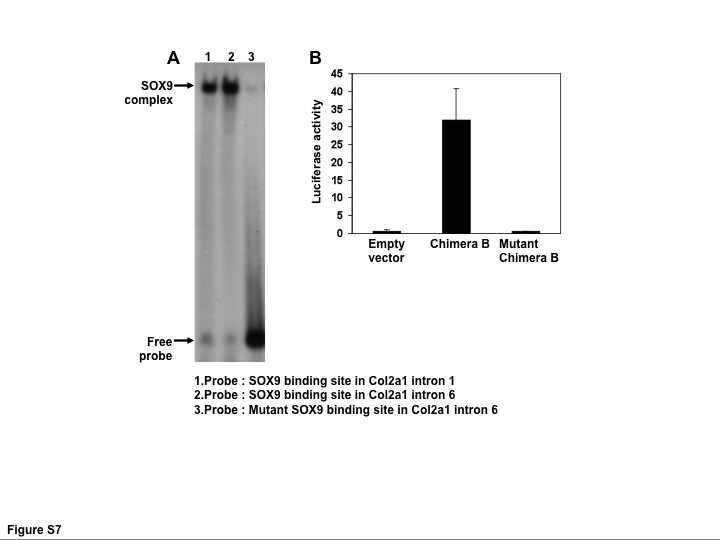

Supplement: Figure S7 — Effect of mutation on the enhancer activity of Chimera B shown in figure 3. The seven bases of 3′ region of the 48bp of intron 6 were mutated as follows. The bases mutated are shown by italics. Wild: CTGGGTTTCTGTAAAGAAGGCCTTCAGCTATCTGA Mutant; CTGGGTTTCTGTCGAAAAGGAAAACAGCTATCTGA The ability of this mutated fragment to bind Sox9 was demonstrated as shown in Figure 2. Lane 1; Control probe (SOX9 binding site of Col2a1 intron 1), lane 2; SOX9 binding site of Col2a1 intron 6, lane 3; mutant SOX9 binding site of Col2a1 intron 6. B. Luciferase reporter assay of Chimera B and mutant Chimera B constructs. By use of this mutant fragment, the mutant Chimera B (Figure 3) construct was prepared and its enhancer activity was compared with wild Chimera B construct using RCS cells. Reporter assay was done as shown Figure 3. The mutant Chimera B did not show the enhancer activity. (1.56 MB TIF) [file pone.0010113.s010.tif]

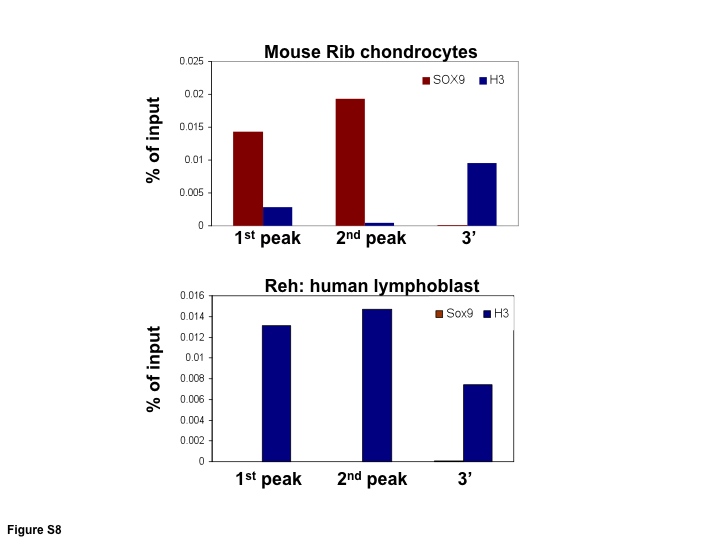

Supplement: Figure S8 — Depletion of histone H3 in 2nd peak of Col2a1 gene in primary chondrocyte. Binding of H3 and Sox9 in Col2a1 gene of mouse rib chondrocyte primary culture and human lymphoblast, Reh cells, was demonstrated by ChIP-qPCR The mouse chondrocytes were cultured as shown previously [68]. The ChIP-qPCR was performed as shown in Figure 4. The primers used in this figure are shown in Table S2. 1st peak and 2nd peak correspond to the peaks in intron 1 and intron 6 of rat Col2a1 gene, respectively. Expression of Col2a1 and Sox9 was detected in primary chondrocyte cells but not in Reh cells by RT-qPCR method shown in Figure S1. The sequence including SOX9 binding site corresponding to intron 6 of the mouse and rat Col2a1 gene is highly conserved in the human Col2a1 gene. (1.56 MB TIF) [file pone.0010113.s011.tif]
